# Curricular course for medical students at a hematology and oncology specialty practice, 2010-2022

**DOI:** 10.3205/zma001561

**Published:** 2022-09-15

**Authors:** Rudolf Weide, Jörg Thomalla, Christoph van Roye, Geothy Chakupurakal, Jochen Heymanns, Hubert Köppler, Stefan Feiten, Jörg-André Nickel, Heinz Schmidberger, Matthias Theobald, Christoph Lutz

**Affiliations:** 1 Praxis für Hämatologie und Onkologie, Koblenz, Germany; 2 Institut für Versorgungsforschung in der Onkologie, Koblenz, Germany; 3 Radiologisches Institut Hohenzollernstraße, Koblenz, Germany; 4 Universitätsmedizin der Johannes Gutenberg-Universität Mainz, Mainz, Germany

**Keywords:** curricular education, academic teaching practice, specialty practice in hematology and oncology, standardized evaluation, small-group teaching

## Abstract

**Aim:** For several years now, medical students have also been taught general practice at academic medical teaching practices. Specialty practices have not yet been included in the curricular education. Since 1998, we have conducted a block seminar in hematology twice per semester for eighth-semester medical students. This block seminar was offered from 1998-2001 to students at the Philipps University in Marburg and since 2001 to students at the Johannes Gutenberg University in Mainz. Since 2010 our block seminar has been part of the curriculum at the Johannes Gutenberg University.

**Method: **Standardized course evaluation by students who had attended our block seminar between January 2010 and March 2022. Courses that were held virtually due to corona were not included in the analysis. The questionnaire used to evaluate courses in the medical degree program at the Johannes Gutenberg University served as the evaluation instrument.

**Results:** Since 1998 more than 1,000 students have attended our seminar. The systematic evaluation of the course by 500 students who participated in the curricular, classroom-based seminar sessions since 2010 shows that the highest ratings possible are given for practical relevance, learning atmosphere, teaching and effectiveness.

**Conclusion: **High quality in teaching curricular courses to medical students at a specialty practice is possible. Insights into the possibilities connected with working in the outpatient setting at a medical practice broadens students’ experience. This teaching format facilitates external university instructors in terms of teaching and, at the same time, relieves the university in terms of staff and financial budget.

## Zusammenfassung

**Zielsetzung: **Die Ausbildung von Studierenden der Humanmedizin im Fach Allgemeinmedizin geschieht seit einigen Jahren auch in akademischen Lehrpraxen. Facharztpraxen sind bisher nicht an der curricularen Lehre beteiligt. Seit 1998 führen wir in unserer Gemeinschaftspraxis zweimal pro Semester ein Blockpraktikum Hämatologie für Studierende der Humanmedizin im 8. Fachsemester durch. Das Blockpraktikum wurde von 1998-2001 für Studierende der Philipps-Universität Marburg und seit 2001 für Studierende der Johannes-Gutenberg-Universität Mainz angeboten. Seit 2010 ist unser Blockpraktikum Teil der curricularen Lehre der Johannes-Gutenberg-Universität Mainz.

**Methodik:** Standardisierte Evaluation der akademischen Lehre durch die Studierenden, die im Zeitraum 01/2010-03/2022 an unserem Blockpraktikum teilgenommen haben. Veranstaltungen, die coronabedingt in virtueller Form stattfanden, wurden in der Analyse nicht berücksichtigt. Als Evaluationsinstrument diente der Fragebogen zur Evaluation der Lehre des Fachbereichs Medizin der Universität Mainz.

**Ergebnisse:** Seit 1998 haben mehr als 1.000 Studierende an unseren Seminaren teilgenommen. Die systematische Evaluation des Unterrichts durch 500 Studierende, die an den curricularen Präsenzveranstaltungen seit 2010 teilgenommen haben, ergibt Bestnoten für Praxisrelevanz, Unterrichtsatmosphäre, Didaktik und Effektivität.

**Schlussfolgerung: **Die curriculare Lehre von Studierenden der Humanmedizin in einer Facharztpraxis ist auf hohem Qualitätsniveau möglich. Einblicke in die Möglichkeiten des ärztlichen Arbeitens im niedergelassenen Bereich erweitern den studentischen Erfahrungshorizont. Durch dieses Lehrmodell wird externen Hochschuldozenten der Unterricht erleichtert, gleichzeitig kann die Universität personell und finanziell entlastet werden.

## 1. Introduction

Since the reform of the German medical licensing regulations (ÄAppO), medical students are not only trained at university hospitals and academic teaching hospitals, but are also trained in general practice at academic teaching practices [https://www.gesetze-im-internet.de/_appro_2002/BJNR240500002.html] [1], [2], [3]. Problem-based learning (PBL) with early connections between theoretical knowledge and real patient interactions has proven itself in medical education not only in the English-speaking countries, but also in Germany and is positively evaluated by students, teachers and patients [4], [5], [6], [7], [8]. As a result, PBL has established itself very widely in Germany, too, over the past 20 years [5], [6], [7], [8]. In particular, the guidance, supervision and feedback from experienced physicians in the role of mentors and teachers has emerged as the central factor in high-quality education [9], [10]. Specialty practices, however, have hardly participated in curricular education in Germany. This is surprising given the exponential growth in medical knowledge and the rapidly advancing medical specialization associated with it.

Since 1998 we have conducted, twice per semester, a block seminar in hematology at our medical practice for students in their clinical semester [11]. The focus of this block seminar is on the differential diagnoses for anemia, polycythemia, leukopenia, leukocytosis, thrombocytopenia, and thrombocytosis. Major intersections with general practice automatically become apparent here. The seminar includes the classification of leukemias and lymphomas, as well as the most important relevant therapeutic principles. The focus of the instruction is on learning from case histories based on existing cases seen in our daily practice. Since 1998 more than 1,000 students have attended our block seminar; and since 2010 at the Johannes Gutenberg University, this elective course – which has been offered for over 12 years – has been part of the eighth-semester curriculum.

The focus of this paper is on the evaluation of the seminar by students since its implementation as a curricular course in 2010 using the standardized evaluation questionnaire used by the Medical School at the Johannes Gutenberg University.

## 2. Methods

Groups of 12 students each were taught by four instructors on a Friday and Saturday for a total of 14 hours. With the start of the COVID-19 pandemic, the seminar has been held three times online using Microsoft TEAMS. The evaluations of the online seminars were not analyzed for methodical reasons. As of the 2021 winter semester, the seminar has been held again in-person in Koblenz.

**Figure 1:**
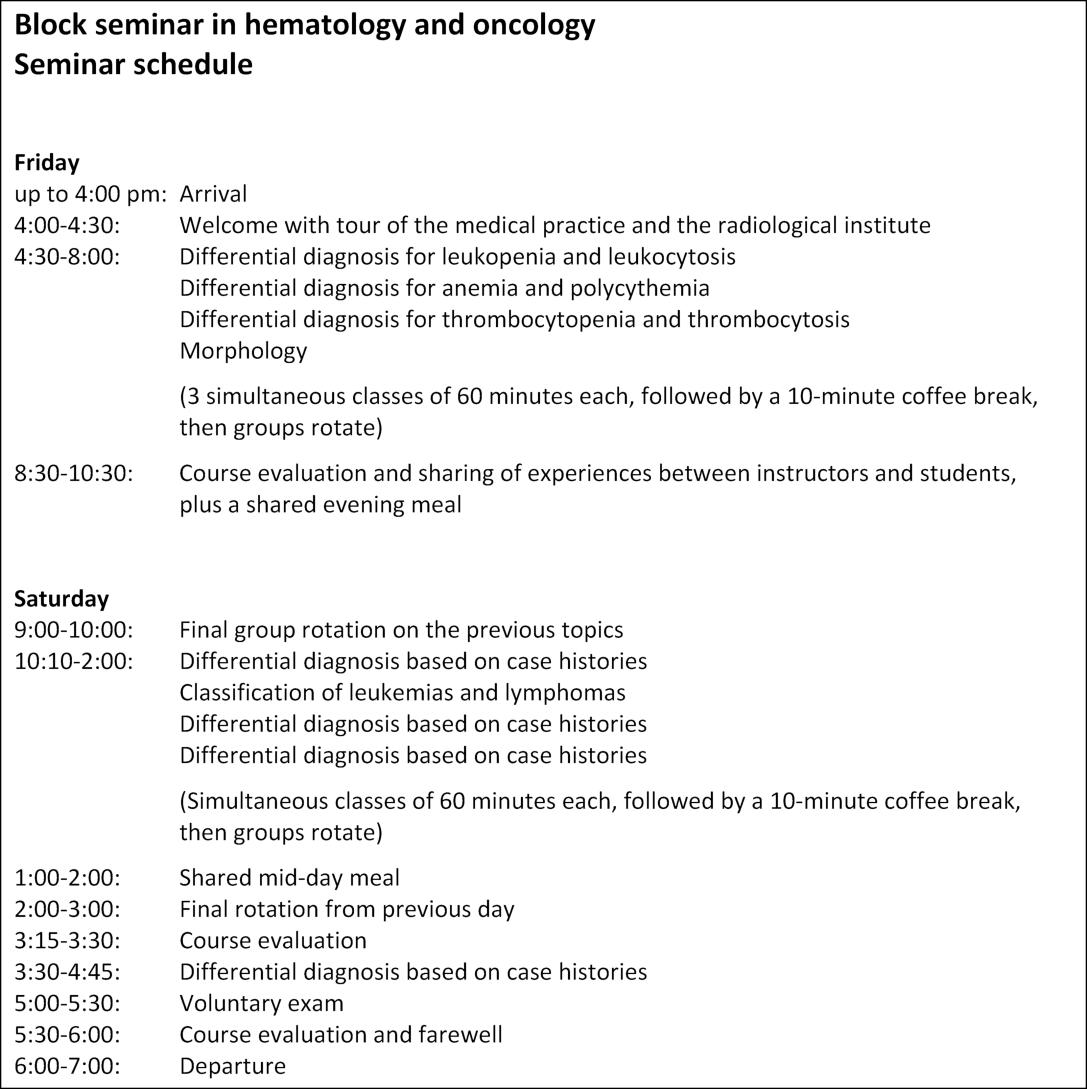
Schedule for the hematology-oncology block seminar

The focus of the instruction is on the differential diagnosis of hematological diseases which is imparted through PBL using case examples. At the end of the seminar, all of the students take a graded oral exam and thus earn academic credit for the Block Practicum in Internal Medicine at the Johannes Gutenberg University. Figure 1 (Fig. 1) provides an overview of the seminar schedule.

At the end of each block seminar, students are asked to evaluate the course using the Johannes Gutenberg University’s standardized questionnaire (see attachment 1 ).

All of the questions are rated by marking a six-point Likert scale (e.g., “Taking this course is worthwhile.” 1=completely disagree; 6=completely agree).

All of the survey results were anonymized, entered into a database and analyzed statistically using SPSS 19.

## 3. Results

More than 1,000 students have taken our block seminar since 1998. The mean age of the students was 26 years (19-55); 69% were female, 31% male.

Since implementing the seminar as a curricular course in 2010, 500 students with an average age of 26 years (19-55) have attended the seminar and evaluated its instruction, of these 73% were female, 27% male.

The reasons given by students for taking the course include: the opportunity to earn academic credit (87%), general interest in the subject matter (56%), recommendation by participating students (11%), instructor (5%) and course’s structure as a block (5%). The mean values, standard deviations and confidence intervals are presented for the different aspects of the evaluation in table 1 (Tab. 1), table 2 (Tab. 2) and table 3 (Tab. 3).

It can be seen that very high ratings were given for the clarity of the teaching objectives and the relevance of the topics covered. The organization of the course content was praised with emphasis on the successful connections between theory and practice. Overall, the teaching objectives were achieved in full, with the scope of the course material not being rated as too wide.

The highest ratings were given for the instructors for their motivation and their inspiring and interesting ways of imparting knowledge. Mentioned in particular were the use of case histories as examples from daily practice and the successful use of teaching aids. From the students’ point of view this can make complicated material more easily understandable and such instruction encourages active and critical engagement with the seminar topics.

High ratings were given for the open, safe and positive learning atmosphere with the opportunity to ask questions and share thoughts. This learning atmosphere also enabled the group, together with the instructor, to solve difficult differential diagnostic questions within the context of case-based learning.

**Figure 2:**
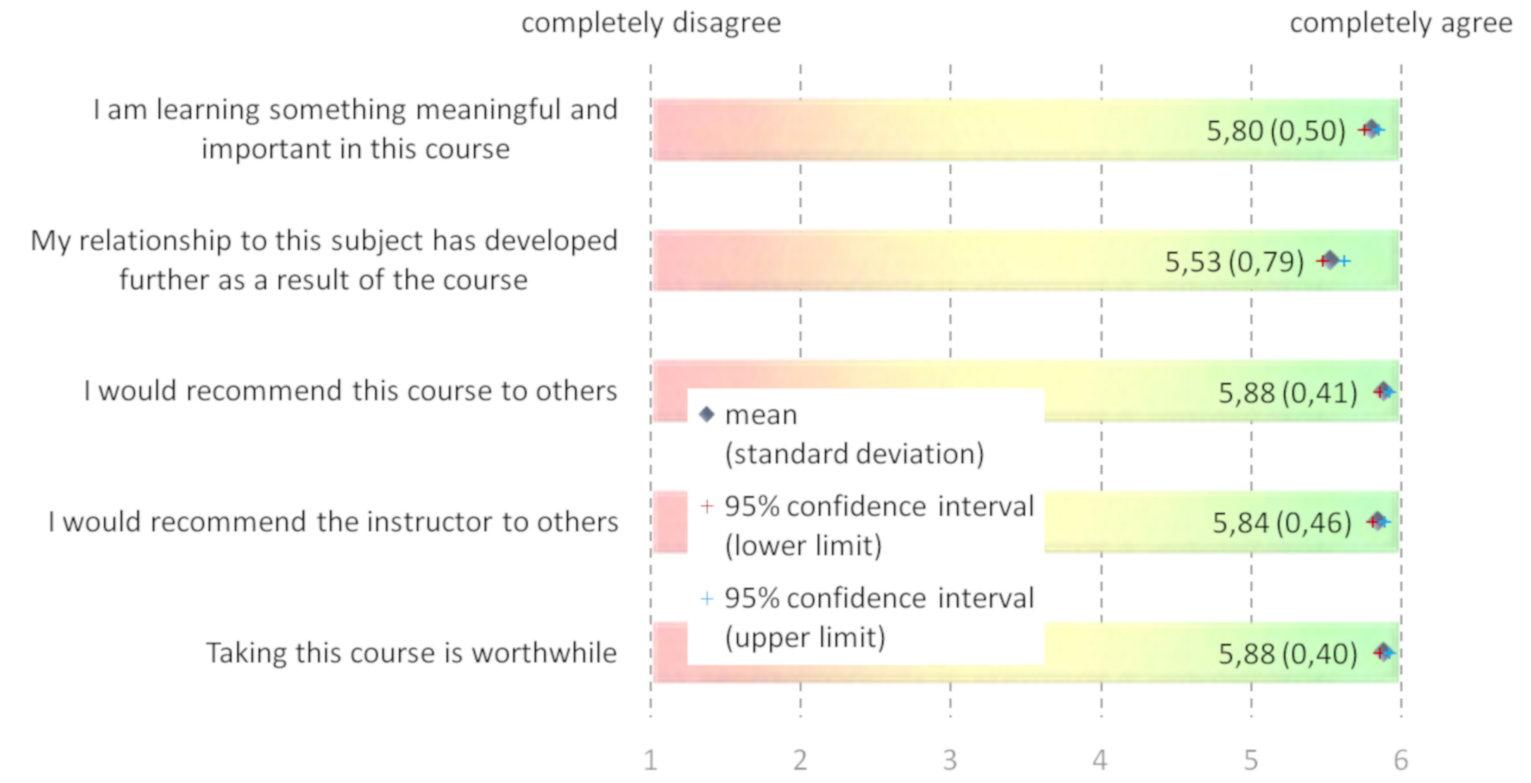
Overall evaluation of the course

All in all, the evaluation results were excellent for objectives and content, teaching, interaction and communication, student participation, and the overall rating of the course (see figure 2 (Fig. 2)).

All of the respondents indicated that taking the course was worthwhile because it enabled them to learn something meaningful and important. Overall, the course and the course instructors were unreservedly recommended by all of the attendees and they reported that their understanding of the subject matter had grown to a great extent.

## 4. Discussion and conclusion

The aim of successful undergraduate medical training should be the mastery of basic theoretical and practical knowledge and communication skills that allow for the medical profession to be practiced under the appropriate supervision. The acquisition of medical competencies, a consistent focus on patients and their needs, the communication between doctor and patient, scholarly work, and the integration of scientific knowledge are all emphasized in the 2020 Master Plan for Medical Study (Masterplan Medizinstudium 2020). The acquisition of knowledge, skills, and exemplary medical conduct are the intended learning objectives [12]. In our opinion, this is only possible if the ambulant sector is included in formal medical studies. In 2019, in Germany 18.8 million patients were treated at hospitals[13] . By contrast, one billion patient interactions took place in the outpatient setting [14]. The enormous and increasing growth in medical knowledge demands, in our opinion, not only the early integration of general practice into the undergraduate medical curriculum, but also, based on the frequency of diseases, exposure to selected medical specialties, such as hematology and oncology, cardiology, gastroenterology, pulmonology, nephrology, infectious diseases, neurology, orthopedics, gynecology, pediatrics, psychiatry and psychotherapy. Such a range of elective courses are already offered at the Goethe University in Frankfurt [15]. Especially in hematology and oncology, the vast majority of patients are diagnosed and treated ambulantly. Alone the practicing hematologists and oncologists in Germany, whose professional association is the BNHO, treat approximately 1,200,000 cancer patients per year [https://bnho.de/ueber-den-bnho/]. Only a small percentage of cancer patients are treated exclusively at university hospitals and university cancer centers. In our opinion, it is essential that cancer diagnostics and therapy in the ambulant sector be included in the medical curriculum during the clinical phase of medical study. The conduction and evaluation of our block seminar as a curricular course for eighth-semester students at the Johannes Gutenberg University shows that such a course held at a specialty practice can be offered at a consistently high level of quality. The systematic evaluation of this seminar by 500 students revealed some of the highest ratings possible for practical relevance, learning atmosphere, teaching and effectiveness. In our view, the standardized questionnaire used by the Johannes Gutenberg University could be improved in several regards, for instance, by asking more nuanced questions to elicit more information on the amount of material to be learned for the course and how well complex ideas are explained by the instructors.

In regard to the necessary rooms and staff at an academic teaching practice in a medical specialty, the requirements for general practice should serve as a role model [2]. There are many arguments for expanding the teaching at academic teaching practices to include medical specialties:

1. As opposed to other European countries, Germany has a widespread and well-functioning network of medical specialists.
2. Many specialist practitioners have many years of experience in medical practice and teaching.
3. Some specialist practitioners have doctorate degrees and are required to fulfill an obligation to teach.
4. Teaching at a practitioner’s own medical practice has many advantages: familiar patient base, rooms, equipment, no travel to and from the university, no time away from the medical practice.
5. It relieves the universities in terms of staff and teaching space.
6. It entails smaller student groups.
7. Insights into the possibilities of working at a medical practice broaden students' experiences.

The definition of small-group instruction as having 6-10 students is, in our opinion, the result of the university's limited resources in staffing and teaching spaces. Small-group instruction should not exceed groups of 3-4 students because personal contact and intensive intellectual interaction between students and instructors create optimal conditions for effective practice-oriented learning. Based on our experience, anxiety about asking questions disappears as a result. A conducive learning atmosphere can be created. Experience stemming from our block seminar shows that with a group size of 3-4 students per instructor students drop their reservations and constructive discussions can ensue. Questions about comprehension and understanding are often asked. This is an indispensable pre-requisite for problem-based learning. Another important curricular aspect is the fact that the students are able to earn academic credit for the seminar held at the medical practice.

In summary, based on our many years of experience with our block seminar, we would like to strongly advocate among universities and medical specialists in support of the idea that there be a comprehensive expansion of this teaching format to include academic instruction at specialty medical practices. We would view it very positively if the curricular education for medical students could be expanded and improved.

## Funding

This paper did not receive any funding from the pharmaceutical industry or from public institutions.

## Competing interests

The authors declare that they have no competing interests. 

## Supplementary Material

Questionnaire for course evaluation

## Figures and Tables

**Table 1:**
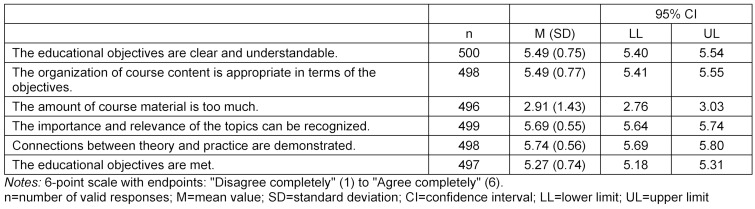
Evaluation of course objectives and content

**Table 2:**
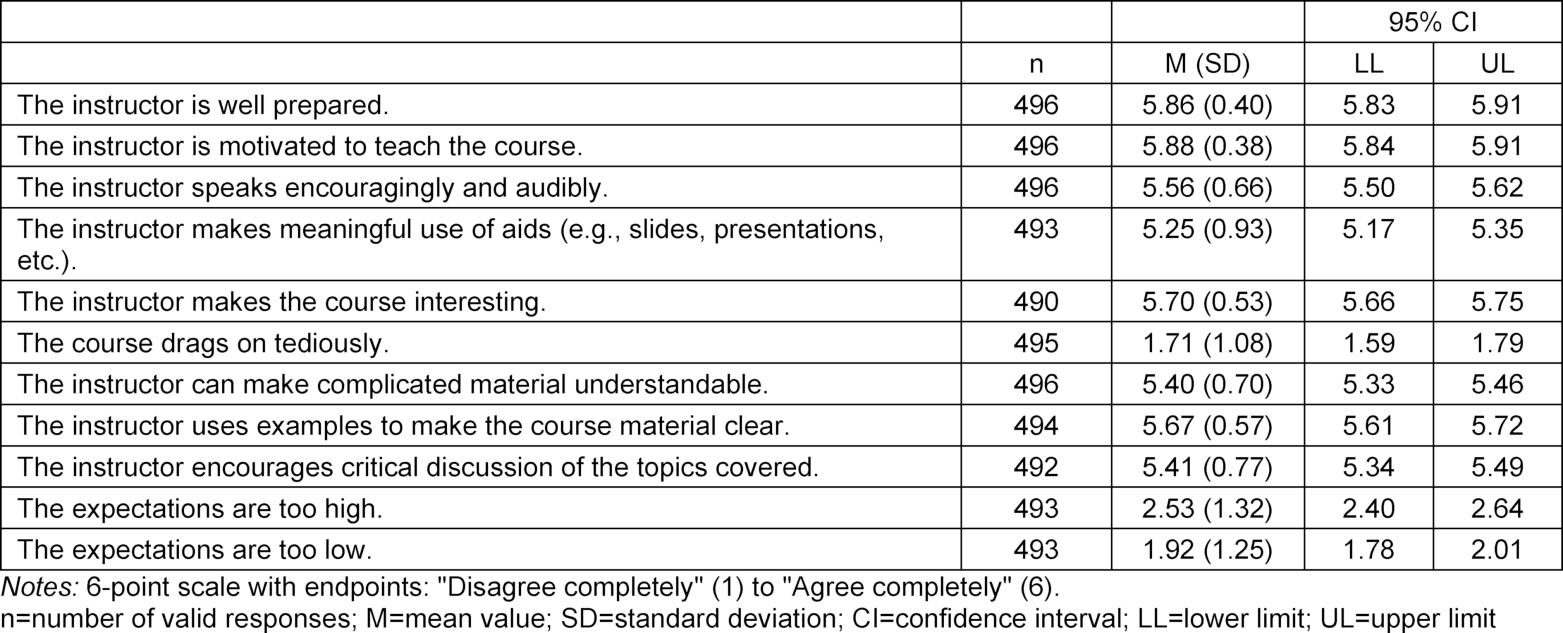
Evaluation of the instructors

**Table 3:**
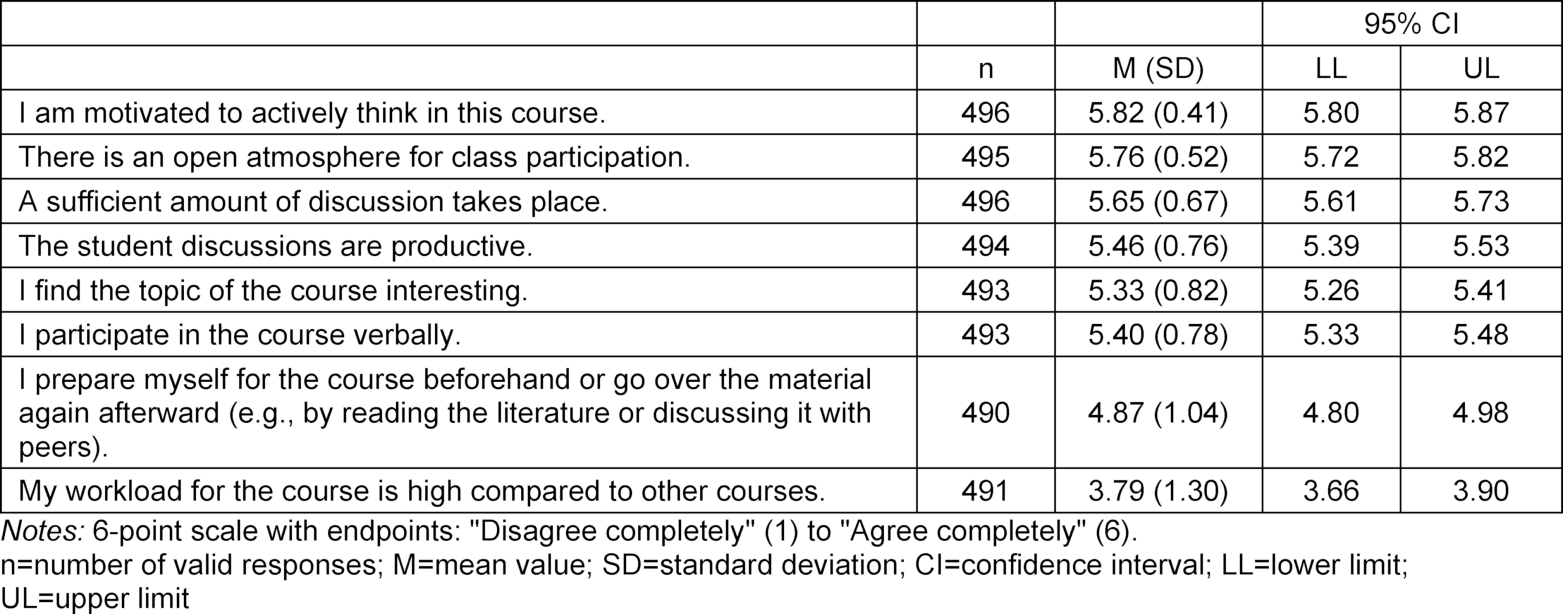
Evaluation of the interaction/communication during the course and the opportunity to participate in the seminar
