# Supplementary material for: Curricular course for medical students at a hematology and oncology specialty practice, 2010-2022
Source: GMS J Med Educ. 2022 Sep 15;39(4):Doc40. doi: 10.3205/zma001561 (PMC9585415; doi:10.3205/zma001561)
Supplement: Questionnaire for course evaluation [file JME-39-40-s-001.pdf]

Course Evaluation

Age: ______ years Sex: ❍ female ❍ male

Semester level: ______

I was absent from the course (please mark the right answer):
❍ 0 times ❍ 1-2 times ❍ 3-4 times ❍ 5-6 times ❍ 7-8 times
❍ more than 8 times

My reason for attending the course (multiple responses possible):
❍ Required course, academic credit, relevant for exams
❍ Instructor
❍ Interest, topic
❍ and/or ____

Please evaluate the course you attended using this survey. Please answer the questions independently and as accurately as possible. You can respond on the scale between "disagree completely" (1) and "agree completely" (6).

Course objectives and content:

		Disagree completely					Agree completely
01	The educational objectives are clear and understandable.	❍	❍	❍	❍	❍	❍
02	The organization of course content is appropriate in terms of the objectives.	❍	❍	❍	❍	❍	❍
03	The amount of course material is too high.	❍	❍	❍	❍	❍	❍
04	The importance and relevance of the topics can be recognized.	❍	❍	❍	❍	❍	❍
05	Connections between theory and practice are demonstrated.	❍	❍	❍	❍	❍	❍
06	The educational objectives are met.	❍	❍	❍	❍	❍	❍

Attachment 1 to Weide R, Thomalla J, van Roye C, Chakupurakal G, Heymanns J, Köppler H, Feiten S, Nickel JA, Schmidberger H, Theobald M, Lutz C. *Curricular course for medical students at a hematology and oncology specialty practice, 2010-2022.* GMS J Med Educ. 2022;39(4):Doc. 40. DOI: 10.3205/zma001561

Evaluation of the instructor:

		Disagree completely					Agree completely
07	The instructor is well prepared.	❍	❍	❍	❍	❍	❍
08	The instructor is motivated to teach the course.	❍	❍	❍	❍	❍	❍
09	The instructor speaks encouragingly and audibly.	❍	❍	❍	❍	❍	❍
10	The instructor makes meaningful use of aids (e.g., slides, presentations, etc.).	❍	❍	❍	❍	❍	❍
11	The instructor makes the course interesting.	❍	❍	❍	❍	❍	❍
12	The course drags on tediously.	❍	❍	❍	❍	❍	❍
13	The instructor can make complicated material understandable.	❍	❍	❍	❍	❍	❍
14	The instructor uses examples to make the course material clear.	❍	❍	❍	❍	❍	❍
15	The instructor encourages critical discussion of the topics covered.	❍	❍	❍	❍	❍	❍
17	The expectations are too high.	❍	❍	❍	❍	❍	❍
18	The expectations are too low.	❍	❍	❍	❍	❍	❍

Interaction/communication during the course and:

19	I am motivated to actively think in this course.	❍	❍	❍	❍	❍	❍
20	There is an open atmosphere for class participation.	❍	❍	❍	❍	❍	❍
21	A sufficient amount of discussion takes place.	❍	❍	❍	❍	❍	❍
22	The student discussions are productive.	❍	❍	❍	❍	❍	❍

Your own participation in the course:

		Disagree completely					Agree completely
23	I find the topic of the course interesting.	❍	❍	❍	❍	❍	❍
24	I participate in the course verbally.	❍	❍	❍	❍	❍	❍
25	I prepare myself for the course beforehand or go over the material again afterward (e.g., by reading the literature or discussing it with peers).	❍	❍	❍	❍	❍	❍
26	My workload for the course is high compared to other courses	❍	❍	❍	❍	❍	❍

Overall evaluation:

27	I am learning something meaningful and important in this course.	❍	❍	❍	❍	❍	❍
28	My relationship to this subject has developed further as a result of the course.	❍	❍	❍	❍	❍	❍
29	I would recommend this course to others.	❍	❍	❍	❍	❍	❍
30	I would recommend the instructor to others.	❍	❍	❍	❍	❍	❍
31	Taking this course is worthwhile.	❍	❍	❍	❍	❍	❍

Comments:

If you still have any comments about the course which you have not been able to share yet, then please use the following table to do so.

Positive aspects	Negative aspects

You have reached the end. Thank you for your cooperation!

Attachment 1 to Weide R, Thomalla J, van Roye C, Chakupurakal G, Heymanns J, Köppler H, Feiten S, Nickel JA, Schmidberger H, Theobald M, Lutz C. *Curricular course for medical students at a hematology and oncology specialty practice, 2010-2022.* GMS J Med Educ. 2022;39(4):Doc. 40. DOI: 10.3205/zma001561
